# Geographic variation in the altitudinal migration patterns, body size, oxidative status and exploratory behavior in a neotropical bird

**DOI:** 10.1002/ece3.9941

**Published:** 2023-03-26

**Authors:** Yanina Poblete, Carolina Contreras, Carolina Fernández, Cristian R. Flores, Patricia Vega, Miguel Ávila, Pablo Sabat

**Affiliations:** ^1^ NIAVA: Núcleo de Investigaciones Aplicadas en Ciencias Veterinarias y Agronómicas, Instituto de Ciencias Naturales, Facultad de Medicina Veterinaria y Agronomía Universidad de Las Américas, Campus Providencia Manuel Montt 948 Santiago Chile; ^2^ Departamento de Ciencias Ecológicas, Facultad de Ciencias Universidad de Chile Santiago Chile; ^3^ Center of Applied Ecology and Sustainability (CAPES) Santiago Chile; ^4^ Facultad de Ciencias Forestales y Conservación de la Naturaleza Universidad de Chile Santiago Chile; ^5^ Liceo Armando Robles Rivera Arauco 474 Valdivia Chile; ^6^ Master of Conservation Science University of Queensland Brisbane Queensland Australia

**Keywords:** altitudinal migration, exploratory behavior, local adaptations, mountains birds, oxidative stress

## Abstract

To cope with life in the mountains, populations of the same species can exhibit substantial variability in their altitudinal migration patterns and phenotypes in response to local weather conditions. Studying such variability can provide valuable insights into how local populations respond to environmental challenges, and this information can be useful for conservation efforts in mountain ecosystems. Here, we used δ^2^H values of feathers and blood to evaluate latitudinal variation in altitudinal migration patterns and its possible links with body size, oxidative status, and exploratory behavior in 72 individuals of rufous‐collared sparrow (*Zonotrichia capensis*) that breed at low and high elevations in the center (~33°) and south (~38°) of Chile. Our results show that both altitudinal migration patterns and oxidative status were significantly influenced by the latitude of breeding sites, while exploratory behavior was associated with elevation. Notably, we found that fast‐explorer birds inhabiting low elevations in central Chile displayed higher levels of oxidative damage than slow‐explorer birds. These outcomes underscore the possibility of local adaptations in response to diverse local environmental conditions in the Andes. We discuss the implications of latitude, elevation, and environmental temperature in shaping the observed patterns and highlight the significance of identifying local adaptations in mountain birds for better predicting their response to climate change and other challenges stemming from anthropogenic activities.

## Graphical abstract

Individuals of the same species can substantially vary their altitudinal migration pattern and phenotype according to local weather conditions. Here, we evaluate latitudinal variation in altitudinal migration patterns and its possible links with body size, oxidative status, and exploratory behavior in a neotropical bird.

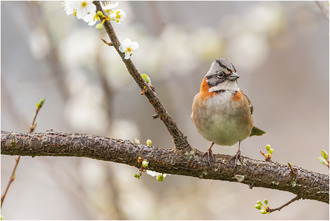

## 1 INTRODUCTION

Mountains cover approximately one fifth of the planet's land surface with a complex topography and environmental gradients changing over short distances as a result of elevation (Körner, 2007). This has favored the evolution of a wide variety of ecosystems and generated high biodiversity, with close to 25% of all terrestrial species dwelling at high elevations (Spehn et al., 2019). In the mountains, approximately 1240 species of birds survive and reproduce with morphological, physiological, and behavioral mechanisms that have been rarely explored in neotropical birds (Barçante et al., 2017; Tsai et al., 2021).

Various bird species perform altitudinal migration, defined as short‐distance seasonal movements between breeding and wintering sites located at different elevations (Boyle, 2017; Faaborq et al., 2010; Rappole, 2013). The benefits of altitudinal migration include high resource availability and suitable sites for both reproduction and wintering (Boyle, 2017; Bridge et al., 2010; Gillis et al., 2008). In contrast, the costs associate to migration may include higher rates of energy expenditure, an increase in the probability of being predated in route and a shorter time for reproduction (Boyle et al., 2016; Hsiung et al., 2018). The main hypothesis explaining the trigger of altitudinal migration is the ‘climatic constraint’ hypothesis, which posits that birds migrate, because there are physiological constraints that limit their abilities to cope with adverse weather conditions (Cox, 1985). Nevertheless, altitudinal migration may also be influenced by a range of ecological factors, including fluctuations in food availability, predator abundance, and inter‐individual variations in dominance (*see* Hsiung et al., 2018 *for detail of these alternative hypotheses*). Such drivers may be particularly significant in tropical environments or when the distance between breeding and wintering elevation is short (Rappole, 2013; Tsai et al., 2021).

Comparative studies about morphology and physiology of mountain birds show that at high elevations, birds often show large body size (Meiri & Dayan, 2003) and down‐regulate their adrenocortical stress response during parental care (Bears et al., 2003; Breuner & Hahn, 2003; Poblete et al., 2020), increases its metabolism (Lindsay et al., 2009; Soobramoney et al., 2003; Weathers et al., 2002), develop a large thermal neutral zone (Martin et al., 1993) and show high‐hematocrit values (Borras et al., 2010) to cope with elevation and weather extremes. As a result of the increased rate of aerobic metabolism at high elevation, it is also likely that birds produce more reactive oxygen species (ROS), which should be counteracted by an increase in antioxidant levels, because an imbalance in intracellular homeostasis between ROS, and antioxidants can lead to oxidative stress (Metcalfe & Alonso‐Alvarez, 2010; Monaghan et al., 2009). Under this condition, organisms suffer cumulative molecular damage, which triggers various diseases and can even cause death (Costantini et al., 2010; van de Crommenacker et al., 2010). A recent study of passerines of the genus *Cinclodes* showed that at high elevations (2.000–5.000 m), *C*. *fuscus* and *C*. *oustaleti* had significantly higher total antioxidant capacity (TAC) than *C*. *nigrofumosus* inhabiting the marine coastal zones (Tapia‐Monsalve et al., 2018). This suggests that altitudinal migrant birds are exposed to higher ROS levels, which are compensated by a higher production of antioxidants. In addition, studies of long‐distance migrants have shown that the physical wear generated by flight produces an increase in ROS and an increase in plasma antioxidant levels (Costantini et al., 2007; Eikenaar et al., 2020). Together, these studies suggest that altitudinal migrant birds should have specific mechanisms to increase antioxidant levels in response to an increase in ROS.

Evidence of behavioral strategies to cope with high‐elevation conditions have also been found. For instance, dark‐eyed junco (*Junco hyemalis*) builds subterranean nests and match the duration of their reproductive period with the period of favorable weather and available food (Bears et al., 2009), and several bird species provide greater parental care to their offspring (Badyaev & Ghalambor, 2001). Under these conditions, birds should also be able to quickly detect the environmental fluctuations to decide when is better seeking food and refuge, migrate, and/or breed (Mettke‐Hofmann et al., 2009; Poblete et al., 2018). Exploratory behavior allows individuals to obtain information about different aspects of the environment (Mettke‐Hofmann et al., 2002). This behavior is usually characterized by the mean activity levels displayed by individuals in response to unfamiliar objects or novel environments (Wilson et al., 1994). Thus, individuals who visit a high number of novel sites, approach closer to unfamiliar objects and show a high movement rate during tests are identified as fast‐exploring, while those showing lower scores in these variables are identified as slow‐exploring (e.g., Dingemanse et al., 2002). Comparative studies on exploratory behavior and migration suggest that long‐distance migrant bird species are more prone to explore novel environments than resident bird species (Mettke‐Hofmann et al., 2009; Mettke‐Hofmann & Gwinner, 2004). Nonetheless, evidence of the relationship between exploratory behavior and altitudinal migration in species with both resident and migrant populations remains scarce and inconclusive. For instance, Kozlovsky et al. (2014) found that *Poecile gambeli* individuals breeding at high elevations visited fewer substrates during a novel environmental test than those living at lower elevations. This suggests that slow explorers could be better able to deal with the more unpredictable environment found at high altitudes since in this situation, trustworthy knowledge and greater flexibility would be needed to make decisions at minimal cost. In contrast, Poblete et al. (2018) found the opposite result in *Zonotrichia capensis*, suggesting the ability to rapidly obtain environmental information via exploration is likely important for funding food or shelter under high‐elevation conditions (see Mettke‐Hofmann et al., 2006).

It is worth noting that both migratory birds and fast‐exploring individuals frequently exhibit higher metabolic rates compared to their resident and/or slow‐exploring counterparts, attributed to their increased activity levels (e.g., Careau et al., 2010; Careau & Garland, 2012; Eikenaar et al., 2020; Huntingford et al., 2010). This is consistent with the ‘Extended Pace‐of‐Life Syndrome’ hypothesis (POLS; Careau et al., 2008; Réale et al., 2010), which proposes that there is a group of interrelated traits that are associated with fast life‐history strategies, such as high‐metabolic rates, short lifespan, high‐reproductive effort, and behaviors like aggression and exploratory behavior. Thus, it is conceivable that both phenotypes are linked as part of a set of local adaptations to life in high‐altitude environments.

Consequently, fast‐exploring individuals may show higher levels of oxidative damage due to their higher metabolic rate than slow‐exploring individuals (Arnold et al., 2015). This is consistent with the idea that higher levels of physical activity, which are often associated with fast‐exploring behavior, can increase the formation of ROS, leading to oxidative damage (e.g., Larcombe et al., 2010). However, the relationship between exploratory behavior and oxidative status is not entirely clear, as a limited number of studies have assessed this relationship and produced mixed results. For example, in mice, less active individuals demonstrated greater antioxidant capacity than more active individuals (Costantini et al., 2008), whereas in birds, low‐activity levels were associated with lower antioxidant capacity and higher ROS levels (Arnold et al., 2015; Herborn et al., 2011).

Collectively, these findings suggest that the variation across environmental gradients can drive selection for diverse life‐history strategies, shaping relationships between phenotypic traits to cope local environmental conditions. In fact, latitudinal temperature and rainfall variation are among the most influential abiotic factors on the ecological conditions experienced by individuals (Hochachka & Somero, 2014). For instance, in areas with highly seasonal climates, birds adjust their timing of breeding and migration to coincide with resource availability (Both & Visser, 2001). Conversely, in temperate areas, birds may time their breeding to coincide with the peak availability of insect prey during the summer months (Visser et al., 2006). Physiological traits such as metabolic rate have also been tested for their effects on latitude, exhibiting a positive association with latitude (e.g., Cavieres & Sabat, 2008; Maldonado, Bozinovic, et al., 2012). Additionally, several studies have reported intraspecific latitudinal variation in behavioral traits, such as sexual behavior (e.g., Botero‐delgadillo, Quirici, Poblete, Ippi, et al., 2020; Petrie & Kempenaers, 1998), competitiveness (e.g., Fujimoto et al., 2015), and nest defense (e.g., Ippi et al., 2013). Thus, assessing the phenotypical variation associated with altitudinal migration between latitudes is probably one of the most appropriate methods to understand altitudinal migration, its subjacent mechanisms, and triggers from an evolutionary perspective (Foster, 1999).

**FIGURE 1:**
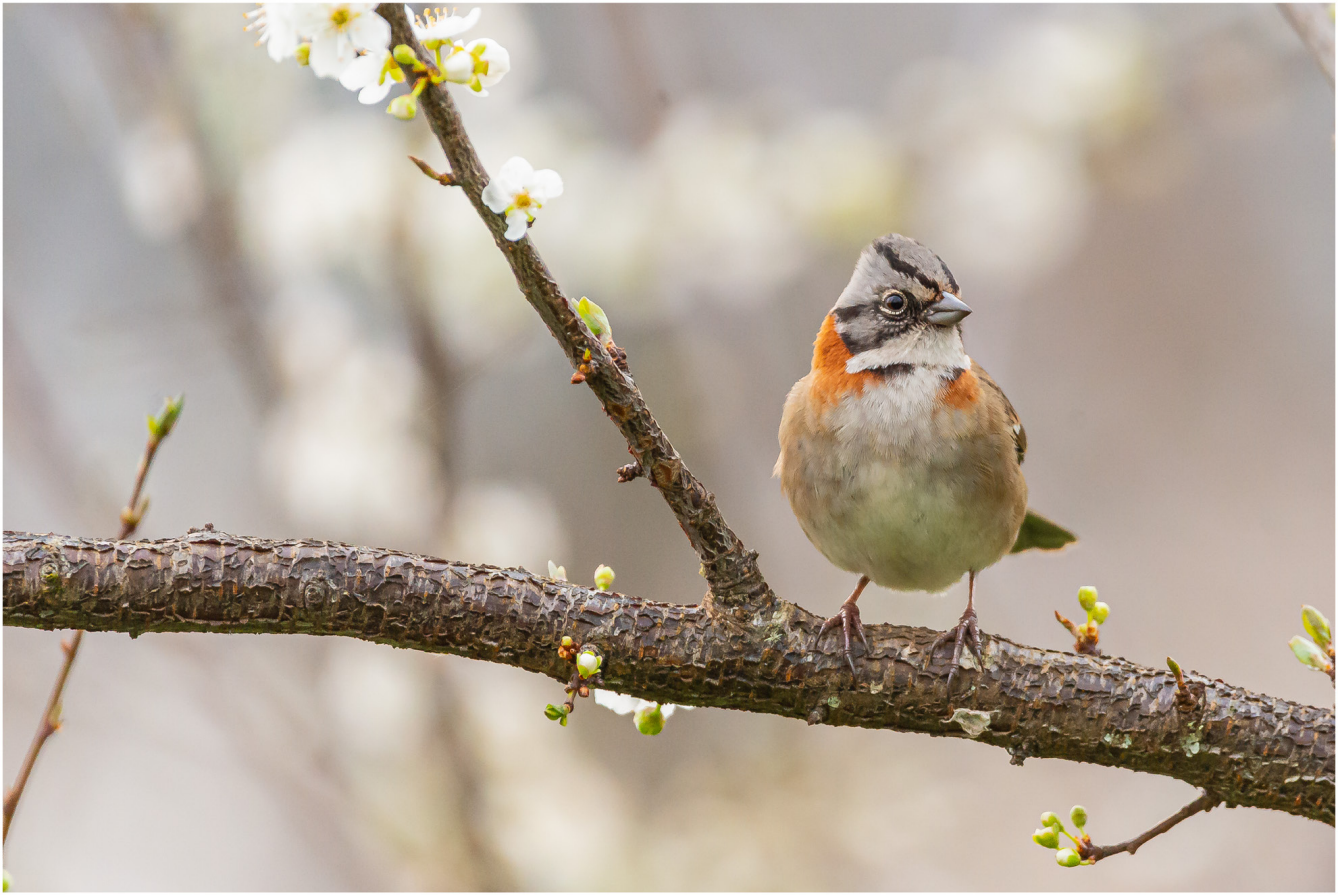
*Zonotrichia capensis*. Photography by Alvaro Huerta.

**FIGURE 2:**
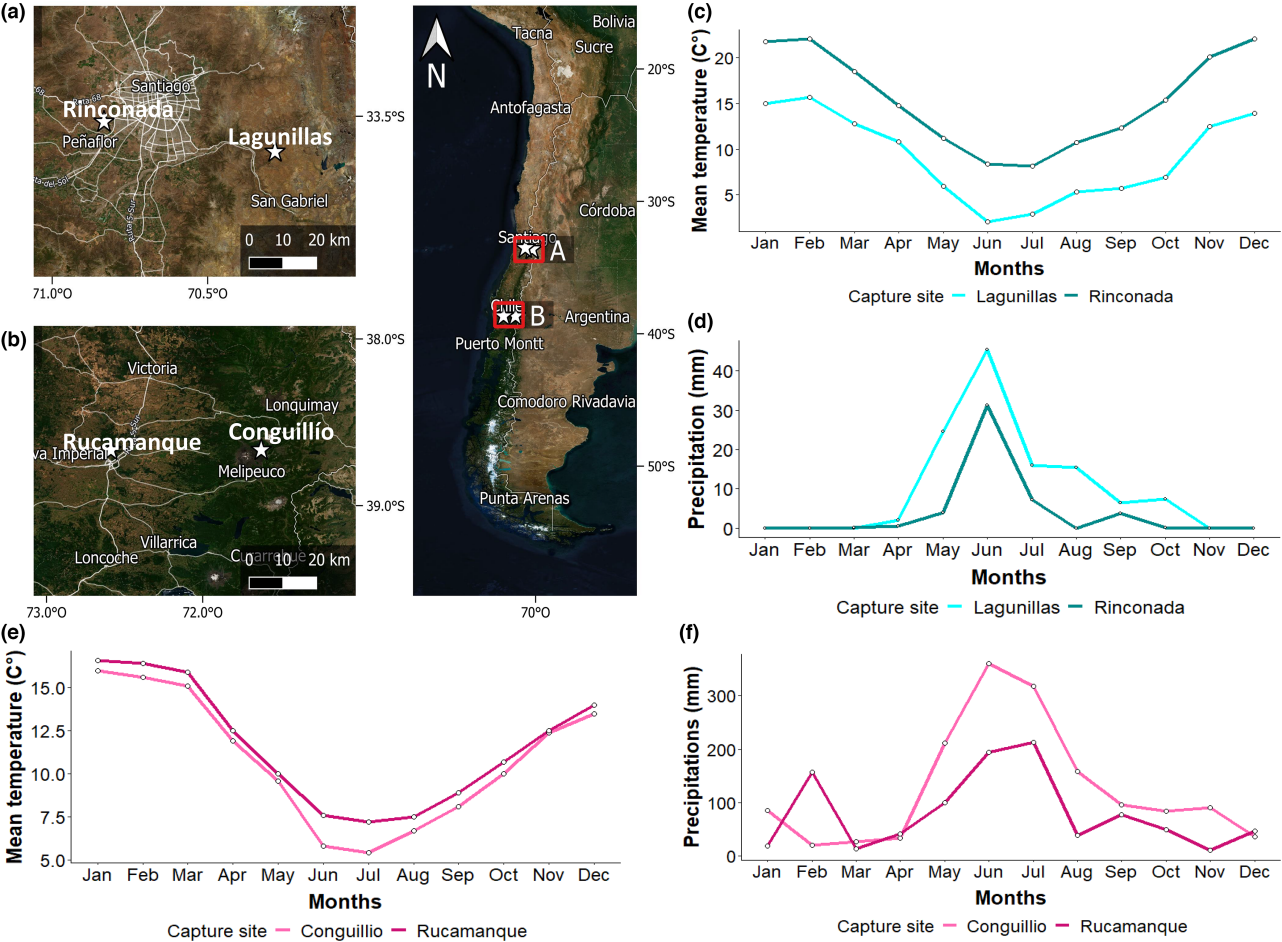
Figure showing the study sites and its weather conditions. The left map shows the four sampled sites: Rinconada (low) and Lagunillas (high) in the center (a) and Rucamanque (low) and Conguillío (high) in the south (b) of Chile (white stars). The right map shows extent of rufous‐collared sparrow's (*Zonotrichia capensis*) distribution in the country (geographical extent of the left panel). Graphics show mean temperature and accumulated precipitation patterns during the study years in the center (2019; c, d) and south (2020; e, f) of Chile.

Here, we characterized altitudinal migration, body size, ROS levels, TAC, oxidative status (ROS/TAC ratio), and exploratory behavior of Rufous‐collared sparrows (*Z*. *capensis*; Figure 1) breeding at high and low elevations in two regions that differ in weather conditions in the center and south of Chile (Figure 2; Mediterranean weather 33°S and Temperate weather ~38°S, respectively). Considering the range of environmental conditions that exists across elevations in the Andes, it is reasonable to hypothesize that breeding birds have developed a range of adaptations that enable them to thrive in their respective local environments. Thus, we predicted that birds breeding at high elevations migrate toward lower elevations during winter, exhibit larger body sizes, have a greater propensity for exploration, and possess higher levels of ROS and TAC than birds that breed at low elevations. Furthermore, we hypothesized that there is a positive relationship between exploratory behavior and oxidative status, as ROS levels and TAC are likely to be influenced by metabolic rate and activity levels. Accordingly, our research objectives were (1) assessing altitudinal migration patterns, (2) comparing the body size, oxidative markers, and exploratory behavior between migrant and resident populations and investigating any differences between study sites, breeding elevation, and latitude, and (3) evaluating the relationship between oxidative status and exploratory behavior among study sites.

## 2 MATERIALS AND METHODS

### 2.1 Study species

Rufous‐collared sparrows is well‐suited species to evaluate differences in body size, oxidative status, and exploratory behavior associated with altitudinal migration because they occupy in a huge variety of environments from southern Mexico (10°N) to the southern tip of South America (55°S) and from sea level to >4000 m of elevation (Chapman, 1940). Variations in the behavior and physiology of rufous‐collared sparrows along latitudinal (Maldonado, Bozinovic, et al., 2012; Sabat et al., 2009; van Dongen et al., 2010) and altitudinal gradients (Poblete et al., 2018, 2020; Ruiz et al., 1995) account for their adaptability to different environmental conditions. Rufous‐collared sparrows are a generalist, omnivorous species that feed mainly on fruits, seeds, and insects (Lopez‐Calleja, 1995). These birds establish their breeding territories during the Austral spring, from September to December at high elevation (see Poblete et al., 2018) and until February at low elevation. The post‐breeding period, from ~January to March, is employed to molt and is followed by the non‐breeding period, from April to August, where some populations moving at low elevations. However, it is important to note that rufous‐collared sparrow may have different timing and duration of their life‐history stages depending on the local environmental conditions (Chapman, 1940).

### 2.2 Study sites

We captured 72 breeding adult individuals of rufous‐collared sparrows using mist nets from four locations that differ in elevation (high >1500; low <500 m) and latitude (central ~33°; south ~38°) in the Southern Hemisphere (Figure 2). At the beginning of breeding season, in September 2019, we sampled in Lagunillas (33°21′S, 70°17′W 2300–2700 m; *n* = 19) and Rinconada de Maipú (33°31′ S, 70°50′ W ~ 450 m; *n* = 21), which are located in the Metropolitan Region (center of Chile; Figure 2a). This area has a Mediterranean climate characterized by dry and hot summers and cold and rainy winters according to the Köppen climate classification (Figure 2c,d; Peel et al., 2007). Historical weather data (*reviewed in* Center for Climate and Resilience Research – CR2; https://explorador.cr2.cl/) reveal that the mean annual precipitation in the lowlands, situated at approximately 500 m.a.s.l., is 219 mm, whereas the high elevations, located at around 2500 m.a.s.l., receive an average annual precipitation of 547 mm. Furthermore, the annual mean temperature at high‐elevation sites is approximately 8.7°C, whereas that in the lowlands is 14.7°C. Natural vegetation is dominated by shrubs and woods of evergreen sclerophyllous species (Donoso, 1982).

In September 2020, we conducted sampling in Conguillío National Park (38°40′S, 71°38′W 1000–1800 m; *n* = 15) and Rucamanque (38°40′S, 72°36′W ~ 400 m; *n* = 17), which are both sites located in the Araucanía Region (south of Chile; Figure 2b). Here, the climate is temperate and characterized by cold and rainy winters, with summer precipitation (Figure 2e,f). Historical weather data (*Reviewed in* Center for Climate and Resilience Research – CR2; https://explorador.cr2.cl/) shows that the lowlands (~400 m.a.s.l.) in this study region receive a mean annual precipitation of approximately 1170.4 mm, while high elevations (~1500 m.a.s.l.) receive 2077 mm. Moreover, the annual mean temperature at high‐elevation sites is found to be approximately 10.7°C, which is only slightly lower than the temperature recorded in the lowlands at 11.2°C. Natural vegetation is dominated by deciduous *Nothofagus* species (e.g., *N*. *obliqua*, *N*. *alessandrii*, *and N*. *glauca*, *Nothofagaceae*; di Castri & Hajek, 1976).

### 2.3 Bird sampling

For each bird captured, we measured their bill, tarsus, wing, and tail length with digital calipers to the nearest 0.1 cm, and we measured body mass with a 60 g balance scale (±0.1 g). These variables were used to obtain a covariate of body size (see below). Before releasing the individuals, we collected a small blood sample (c. 50–100 μL) from the brachial vein using heparinized tubes, which were stored on ice (4°C) for a maximum of 5 h before reaching the laboratory. We also stored a blood sample (c. 20 μL) in FTA cards (Whatman, Buckinghamshire, UK) for molecular sexing by amplifying the CHD locus using the primers 2550F (5′‐GTTACTGATTCGTCTACGAGA‐3′) and 2718R (5′‐ATTGAAATGATCCAGTGCTTG‐3′) (Fridolfsson & Ellegren, 1999; see Appendix S1 for details). Finally, a second set of aliquots of blood were dried on two glass microscope slides and a primary feather (p9) was collected from seven individuals randomly picked in each study site to analyze the proportions of stable hydrogen isotopes (δ^2^H).

### 2.4 Stable isotope analysis describing altitudinal migration

In mountainous regions, the relationship between δ^2^H and elevation can vary depending on factors such as topography, seasonality, and proximity to moisture sources. However, it is generally accepted that there is a decrease of approximately 0.5‰ in δ^2^H values for every 100 m increase in elevation (Gonfiantini, 1986; Newsome et al., 2015). Here, we compared δ^2^H present in primary feathers (p9) and blood to detect altitudinal migration during the annual cycle of the birds (Newsome et al., 2015; Poblete et al., 2018; Villegas et al., 2016). The primary feather (p9) is produced annually after the breeding stage and prior to migration in the study species (see Poblete et al., 2018), providing a record of the bird's breeding sites. Furthermore, this feather is a convenient sampling material for isotopic analysis, owing to its ease of collection without causing harm to the bird and its substantial quantity of material for analysis. We also used whole blood, which has a renovation rate of ~1 months, and hence, the δ^2^H values of the blood samples collected in September (onset spring) represent the elevations occupied by the birds during the winter months (July and August; Poblete et al., 2018).

Primary feathers were cut into small (<1 mm) pieces, air‐dried and placed in a 2:1 chloroform:methanol solvent mixture to remove surface contaminants. Then, we scraped the whole dried blood samples off the slides, put them into microcentrifuge tubes, and homogenized them by mixing. δ^2^H values were measured with a Thermo Scientific high‐temperature conversion elemental analyzer coupled to a Thermo Scientific Delta V isotope ratio mass spectrometer at the Center for Stable Isotopes at the University of New Mexico. Isotopic results are expressed as d values, δ^2^H = 1000 × [(R_sample_ − R_standard_/R_standard_)], where R_sample_ and R_standard_ are the ^2^H/^1^H of the sample and the standard, respectively. Vienna Standard Mean Ocean Water (V‐SMOW) is the internationally accepted standard for ™^2^H analysis, and the units are parts per thousand or per mil (‰). Precision for δ^2^H was determined by analysis of the three exchangeable internal reference materials for each tissue (blood or feather); the within‐run variation (SD) in δ^2^H values of these reference materials was 3–4‰.

### 2.5 Oxidative status

We centrifuged the blood samples collected in heparinized tubes at 7871 *g* and then separated the plasma and froze it at −80°C until measurement of ROS and TAC (Sabat et al., 2017; see below). We used the TAC as an indicator of non‐enzymatic molecular antioxidants and thiobarbituric acid‐reactive substances (TBARS) as an indicator of oxidative damage (Fernández et al., 2021; Sabat et al., 2017), as it has been done in other bird species (e.g., Gutiérrez et al., 2019; Tapia‐Monsalve et al., 2018). Plasma TAC levels were determined using the antioxidant capacity reduction method (Apak et al., 2006; Ribeiro et al., 2011). The assay evaluates the reduction of the copper (II)‐neocuproine complex to the copper (I)‐neocuproine complex by antioxidants present in the plasma. This reaction can be measured by colorimetry at 450 nm. Finally, the sample value was compared with a Trolox standard curve (Ribeiro et al., 2011). The level of TBARS was estimated using the thiobarbituric acid (TBA) concentration based on a reaction that evaluates the 1:2 adduct formed by malondialdehyde (MDA; a product of lipid peroxidation) and TBA. The MDA‐TBA adduct was determined to have 532 nm colorimetry (Ohkawa et al., 1979). Finally, the oxidative status of individuals was estimated using the TBARS/TAC ratio, with higher values indicating higher oxidative stress (Gutiérrez et al., 2019).

### 2.6 Exploratory behavior

To evaluate exploratory behavior, we conducted novel environmental tests shortly after capturing the birds, following the protocol described by Verbeek et al. (1996). To this end, we used a field‐portable aviary (270 cm long × 150 cm wide × 150 cm high) made of removable poles and semitransparent black cloth (see Poblete et al., 2018, 2021; Botero‐delgadillo, Quirici, Poblete, Poulin, et al., 2020 for aviary details). The aviary had 14 possible perching locations, including perches and walls. Before the trial, the birds had a five‐minute acclimatization period in a small cage (30 cm long × 25 cm wide × 39 cm high) placed in a corner of the aviary and covered with a black cloth. About 1 min before starting the trial, we removed this cloth, and the door of the acclimatization cage was opened for each subject who was free to explore. We used a digital camera (Sony DCR‐68) for recording the behavior of each bird for 10 min (Botero‐delgadillo et al., 2019; Poblete et al., 2018, 2021).

We analyzed the footage using J‐Watcher software. The frequency of visits in each of the 14 areas was used to calculate the Brillouin diversity index, which was used as an indicator of ‘exploratory diversity’ (Blumstein & Daniel, 2007; Botero‐delgadillo et al., 2019; Poblete et al., 2018).

### 2.7 Statistical tests

To mitigate the risk of overparameterization arising from our limited isotopic sample size and the numerous variables involved, we employed distinct models for each of our three objectives. First, to evaluate the altitudinal migration patterns, we used repeated‐measures ANOVA with elevation (high and low) as factor and δ^2^H values of each tissue as repeated measures within each individual, to compare feather (representing summer) and blood (representing winter) δ^2^H values among birds breeding at different elevations in the center and south of Chile and used a post hoc Tukey test to assess for specific differences among means in δ^2^H values of both tissues between seasons and elevations. To this end, we corrected the blood δ^2^H values using the δ^2^H_feather‐blood_ offset of +24‰ observed in *Cinclodes nigrofumosus*, a passerine bird dwelling in coastal sites all year‐round in Chile, to represent tissue specific differences in ^2^H discrimination between blood and feather (*P*. *Sabat unpublished data*). A similar, but of larger magnitude, δ^2^H_feather‐blood_ offset was observed in quails (+38‰, Wolf et al., 2012).

Second, we used nested ANOVA and post hoc Tukey test to compare the body size, oxidative markers, and exploratory behavior between migrant and resident populations and to assess differences between study sites, breeding elevation, and latitude. Here, we fitted separate models that included body size, physiological (TAC, TBARS levels, and ROS/TAC ratio), and behavioral (exploratory diversity) data as the response variables and population status (migrant or resident) and study site nested in elevation (‘high’ or ‘low’) and latitude (‘center’ or ‘south’) as predictors. Given that both body size and sex affect the physiology (e.g., Markó et al., 2011) and behavior of adult birds (e.g., Kelleher et al., 2017; van Overveld et al., 2014), we preliminarily fitted linear models for each response variable and included sex and body size index (BSI) (i.e., the first principal component obtained from principal component analysis (PCA) between morphological variables; see Table S1 for PCA details; Grant & Grant, 2008; Weeks et al., 2020) as predictors (see Table S2 for models). BSI and sex were included as covariates and cofactors in the predictive models, only if their effects on the response variable were statistically significant to avoid overparameterizing the models.

Finally, we used Linear Mixed Model (LMM) with a Gaussian error distribution and log‐link function to evaluate the relationship between oxidative status and exploratory behavior. To this end, we fitted separate models that included TAC, TBARS levels, and ROS/TAC ratio as response variables, exploratory behavior nested in study sites as predictor variables and elevation nested in latitude as a random effect. For all analyses, we checked and validated the model assumptions. Box Cox transformation (*x* ^*λ*^ *−*1)/(λ) with *λ* = −1.595 used to fit thess TBARS levels and ROS/TAC ratio to a normal distribution. We considered an effect to be statistically supported when *p* < .05 and the 95% CI around the estimate did not overlap zero. All analyses were performed in the R Studio statistical environment v.1.4.17.17 using the Base (version v.1.4.17.17), stats (version 3.6.2) and lme4 (version 1.1.‐27.1) packages.

## 3 RESULTS

**FIGURE 3:**
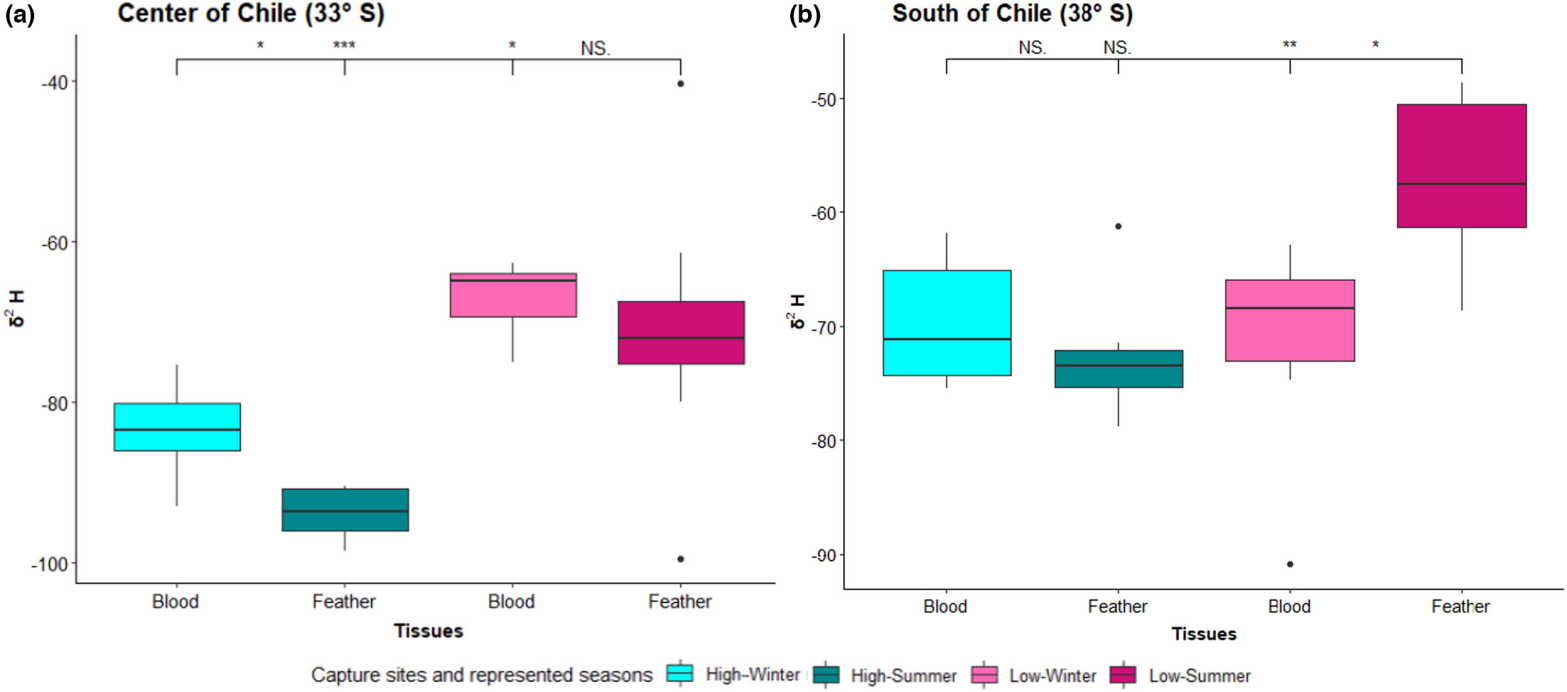
Whole blood and feather δ^2^ H (mean ± SE) values in *Z*. *capensis* in birds that breeding at high and low elevations in the Center (a) and South (b) of Chile. The dates show significant difference between feather δ^2^H values (representing summer) between birds that breeding at high and low elevation in each latitude. Comparison of blood δ^2^H values (representing winter) between elevations show significant differences only in central zone. Significant difference between blood δ^2^H values (representing winter) and feather δ^2^H values (representing summer) were found at high elevation in the central zone and at low elevation in south zone. The bold horizontal line inside the box represents the median δ^2^H values. Vertical lines indicate standard error and asterisks indicate significant differences among groups.

In the center of Chile, the δ^2^H values of blood representing winter collected from high elevation (−84 ± 2‰) were significantly lower to δ^2^H values of blood collected at the same time from sparrows at low elevation (−67 ± 2‰; Tukey: *p* < .001; Figure 3a). The δ^2^H values of feathers representing summer collected from high elevations (−95 ± 2‰) had significantly lower δ^2^H values than feathers collected at the same time from sparrows at low elevations (−67 ± 2‰; Tukey: *p* < .001; Figure 3a). The δ^2^H values of blood samples representing winter (−86 ± 2‰) collected from high elevation were significantly lower than δ^2^H values of feather representing summer collected at the same elevation (−95 ± 2‰; *F* = 9.96; *p* > .05; Figure 3a). The δ^2^H values of blood samples representing winter (−67 ± 2‰) collected from low elevation were similar to δ^2^H values of feather representing summer collected at the same elevation (−67 ± 2‰; *F* = 9.96; *p* > .05; Figure 3a).

In the south of Chile, we found that δ^2^H values of blood samples representing winter (−70 ± 2‰) collected from high elevation were similar to δ^2^H values of blood collected at the same time from sparrows at low elevation (−72 ± 2‰; *p* > .05; Figure 3b). The δ^2^H values of feathers representing summer collected from high elevations (−73 ± 2‰) had significantly lower δ^2^H values than feathers collected at the same time from sparrows at low elevations (−57 ± 2‰; Tukey: *p* < .01; Figure 3b). The δ^2^H values of blood samples representing winter (−70 ± 2‰) collected from high elevation were similar to δ^2^H values of feather representing summer collected at the same elevation (−73 ± 2‰; *p* > .05; Figure 3b). The δ^2^H values of blood samples representing winter (−72 ± 2‰) collected from low elevation were significantly lower than δ^2^H values of feather representing summer collected at the same elevation (−57 ± 2‰; *F* = 9.96; *p* < .05; Figure 3b).

**FIGURE 4:**
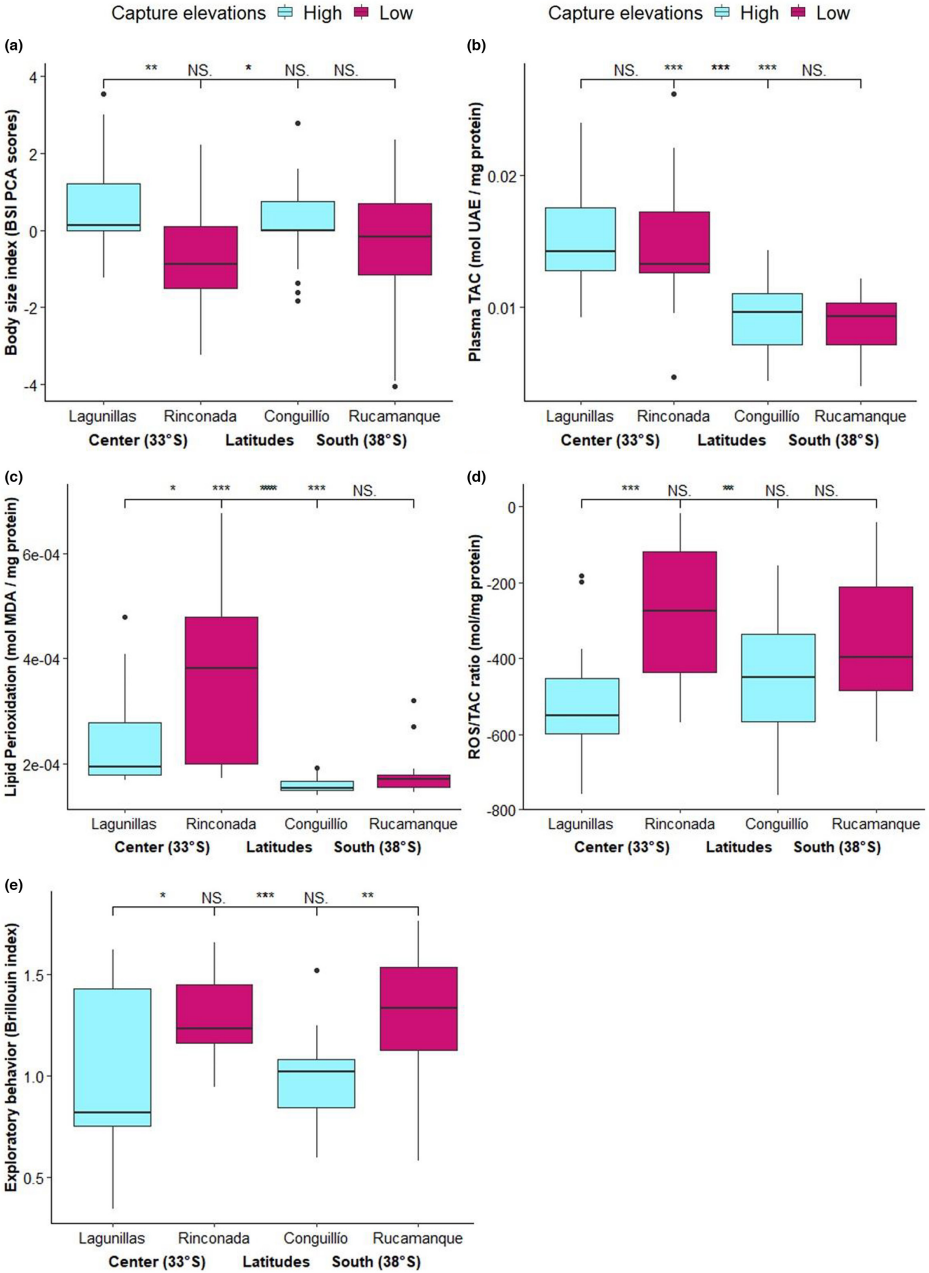
Differences in body size, TAC, TBARS, oxidative status, and exploratory behavior between populations of Rufous‐collared sparrow located at high and low elevations in the center and south of Chile. The bold horizontal line inside the box represents the median score for body size (a) TAC (b), TBARS (c), oxidative status (d), and exploratory behavior (e), respectively. Vertical lines indicate standard error and asterisks indicate significant differences among groups.

Regardless of differences observed in altitudinal migration patterns, we did not find significant differences in body mass, TAC, TBARS levels, oxidative status, or exploratory behavior associated with migrant or resident population status (Table 1). However, we found significant differences in these variables among breeding elevations, study sites, and latitudes (Table 1; Figure 4). Specifically, in the center of Chile, the birds that breed at high elevation were significantly larger than those that breed at low elevations (Figure 4a), but in the south of Chile, we did not find significant differences in body size associated with the elevation of the breeding sites (Figure 4a; see Table S3 for pairwise comparisons).

**TABLE 1:** Results from nested ANOVAs for testing relationship between variation in Body size, TAC, TBARS, oxidative status and exploratory behavior in Rufous‐collared sparrow (*n* = 72).

	df	*F* value	*p*
Effect on BSI			
Population status (migrant or resident)	1	1.859	.176
Population: Elevation: Latitude	2	5.099	.008**
Residuals	84		
Effect on TAC			
Population status (migrant or resident)	1	0.122	.728
Population:Elevation:Latitude	2	23.652	2.04e‐^08^***
Residuals	64		
Effect on TBARS			
Population status (migrant or resident)	1	1.83	.237
Population:Elevation:Latitude	2	26.61	4.91e^10^***
Sex	1	11.37	.001**
Residuals	64		
Effect on TBARS/TAC ratio			
Population status (migrant or resident)	1	3.506	.066
Population:Elevation:Latitude	2	6.624	.002**
Residuals	64		
Effect on exploratory diversity			
Population status (migrant or resident)	1	0.16	.699
Population:Elevation:Latitude	2	4.78	.011*
BSI	1	12.36	.001**
Residuals	64		

*Note*: Aesthetics indicate significant effects.

*p < .005, **p < .01 and ***p < .001, respectively.

Plasma levels of TAC and TBARS were significantly higher in birds from central Chile than from the south (Table 1; Figure 4). In the center of Chile, TAC values were similar between birds from different elevations (Figure 4b), but birds that breed at low elevation showed TBARS levels higher than those that breed at high elevation (Figure 4c). In the south of Chile, we did not observe any differences in TAC and TBARS levels between elevations (Figure 4b,c; see Table S3 for pairwise comparisons). Regarding the ROS/TAC ratio, we found that birds that breed at low elevations in the center of Chile showed significantly higher signal of oxidative damage (Figure 4d see Table S3 for pairwise comparisons).

**FIGURE 5:**
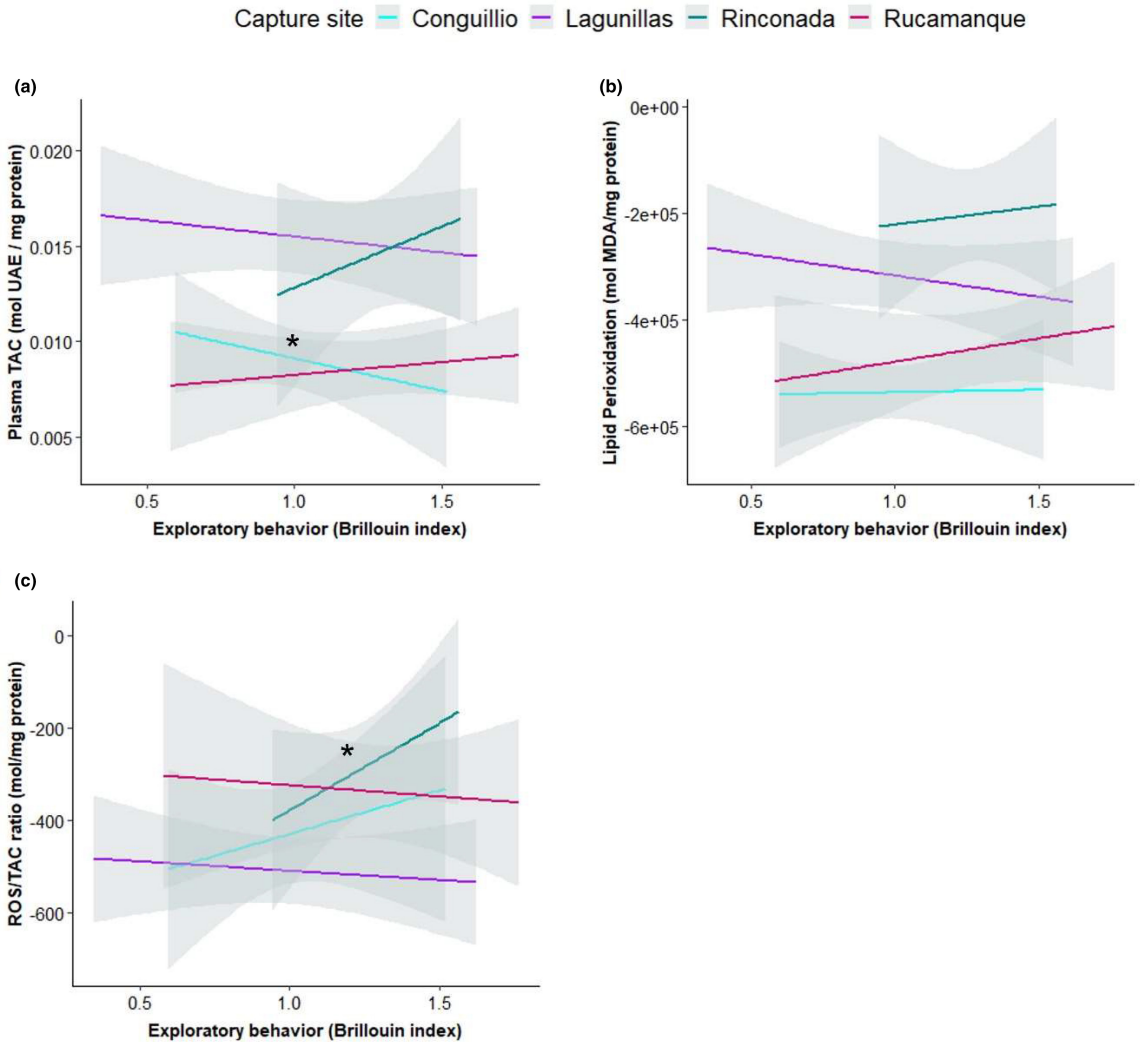
Relationships between TAC (a), TBAR (b) and oxidative status (c) with exploratory behavior in four populations of Rufous‐collared sparrow. Shown model predictions with 95% confidence intervals (lines and shaded area). Asterisks indicate intervals that did not include zero.

The exploratory diversity index was significantly higher in the birds breeding at low elevations, regardless of latitude and study site (Table 1; Figure 4e; see Table S3 for pairwise comparisons). Finally, we found a positive and significant relationship between exploratory diversity with TAC levels in Conguillío (Figure 5a) and with ROS/TAC ratio in Rinconada (Table 2; Figure 5c). We did not find significant relationships between TBARS levels and exploratory behavior (Table 2; Figure 5b).

**TABLE 2:** Mixed‐effects linear models showing the relationships between TAC, TBARS and oxidative status levels with exploratory behavior in Rufous‐collared sparrow (*n* = 72).

	Estimate	SE	L 95% CI	U 95% CI
**Effect on TAC**				
Intercept	0.011	0.005	**2.018** ^**e‐03**^	**2.070** ^**e‐02**^
Exploratory diversity: Conguillío	−0.006	0.002	**−1.069** ^**e‐02**^	**−2.099** ^**e‐03**^
Exploratory diversity: Lagunillas	−0.004	0.002	−4.043^e‐03^	3.738^e‐03^
Exploratory diversity: Rinconada	0.005	0.003	−2.32^e‐04^	1.059^e‐02^
Exploratory diversity: Rucamanque	0.001	0.003	−4.944^e‐03^	5.733^e‐03^
**Random effect**				**σ** ^**2**^
Latitude: Elevation				1.633^e‐15^
**Effect on TBARS**				
Intercept	−550,505	138,153	**−828685.4**	**−270373.8**
Exploratory diversity: Conguillío	−102,428	168,959	−468138.0	240738.2
Exploratory diversity: Lagunillas	−61,169	98,533	−253321.5	141509.6
Exploratory diversity: Rinconada	180,163	153,059	−177430.1	526933.4
Exploratory diversity: Rucamanque	39,293	116,069	−219309.5	279116.7
**Random effect**				**σ** ^**2**^
Latitude: Elevation				3.755^e+10^
**Effect on oxidative status**				
Intercept	−469.89	81.66	**−625.887**	**−313.676**
Exploratory diversity: Conguillío	45.30	91.82	−130.116	220.661
Exploratory diversity: Lagunillas	−38.87	80.40	−192.462	114.725
Exploratory diversity: Rinconada	153.80	73.17	**14.005**	**293.488**
Exploratory diversity: Rucamanque	94.10	68.02	−35.991	224.062
**Random effect**				**σ** ^**2**^
Latitude: Elevation				0.000

*Note*: Bold numbers indicate intervals that did not include zero.

Abbreviations: L/U 95% CI = lower/upper bound; SE, standard error.

## 4 DISCUSSION

In this study, we observed variations in altitudinal migration, body size, oxidative status, and exploratory behavior of *Zonotrichia capensis* across different elevations and latitudes of the study sites. Our stable isotope analysis revealed that *Z*. *capensis* from central Chile, bred at high elevations (~2500 m) and migrated downslope during the winter months, while those breeding at low elevations (~500 m) remained at the same elevation throughout the year (Figure 3a). Similar observations have been reported in the rufous‐collared‐sparrow, which breeds at high and low elevations in sites nearby our study areas (Poblete et al., 2018). In the south of Chile, on the other hand, we found that birds that breed at high elevations remained at high elevations year‐round (~1500 m), while birds that breed at low elevations (~400 m) moved upslope during the winter months.

The ‘climatic constraint’ hypothesis predicts that altitudinal migration is a behavior forced by harsh weather conditions that occur at high elevations during winter (Cox, 1985). Our results support this hypothesis and may explain the pattern observed in the center of Chile because climatic variability between seasons increased with elevation (Figure 2c). Based on data of The Center for Climate and Resilience, during the winter months the breeding site at high elevations was cool and rainy, while in the summer, the temperature increased, and precipitation considerably decreased (Figure 2c,d). Interesting, the wintering sites located at ~1000 m.a.s.l. in the center of Chile show precipitation and temperature conditions similar to those at high‐elevation site during breeding season (~1000 m.a.s.l. winter: mean temperature: 11.8°C; mean accumulated precipitation: 13.0 mm) suggesting that the birds move between elevations to maintain year‐round under certain weather conditions. At low‐elevation sites (~500 m), where birds are resident, the mean temperature was between 10.9°C and 21.0°C and precipitation did not exceed 4.6 mm year‐round (Figure 2d). Some bird species can reside in an area throughout the year when the environmental factors of that area meet their requirements. For instance, if a bird species is adapted to the climate and food resources of a particular location and those resources are available year‐round, then there may be no need for the birds to migrate (Rappole, 2013). A previous study performed in this area shown that 83% of raptor species are resident birds that breed and wintering in central Chile (Jaksic et al., 2001) and ebird.org data show that several passerine bird species such as *Troglodytes aedon*, *Diuca diuca*, *Spinus barbatus*, *Scytalopus fuscus* can be found year‐around in this location. These findings suggest that the temperature and precipitation range in this area may not be the primary drivers of the evolution of altitudinal migration in this area.

In the south of Chile, on the other hand, birds breeding at high elevations appear to exhibit minor seasonal movement by moving slightly downslope during winter. This short altitudinal movement between seasons, however, did not have statistical significance. Instead, the birds that breed at low elevations move upslope during winter. This pattern does not support the ‘climatic constraint’ hypothesis, possibly because during the winter and summer months, the mean temperatures between elevations are similar (Winter: high = 7.5°C, low = 8.9°C; Summer: high = 14.1°C, low = 13.8°C), and both sites are rainy year‐round (Winter: high = 211.37 mm, low = 100.66 mm; Summer: high = 84.8 mm, low = 30.4 mm). Thus, it is possible that weather variation in temperature and precipitation in this region is not sufficient to drive altitudinal migration. Some birds also migrate to higher elevations to avoid competition or predation from other species or to find more suitable habitats for their specific needs (Rappole, 2013). The microclimates of high‐elevation habitats can also provide more stable temperatures and shelter from harsh winter conditions (Hsiung et al., 2018). In additon, it is possible that some birds move downslope for breeding as a strategy to reduce intraspecific competition for territories and sites appropriated for nesting. The ‘dominance’ hypothesis predicts that migrating individuals would be subdominant and hence excluded during the competition for high‐quality habitats with better nesting sites (Gauthreaux, 1978; Marra, 2000). Usually, subdominant individuals are smaller than dominant individuals (e.g., French & Smith, 2005). Here, we did not find significant differences in BSI between birds that breed at high and low elevations in this region, but we observed that at low elevations, the birds tended to be smaller than those that breed and remain at high elevations during winter (see Figure S1 for comparison between all morphometric measures). This result suggests that the dominance hypothesis might partially explain the pattern observed.

Our results also show that in the center of Chile, birds at high elevations were significantly larger than those breeding at low elevations. This result is similar to that shown in a previous study comparing morphological measures between birds breeding at high and low elevations in this region (Poblete et al., 2018). However, as stated above, in the south of Chile, the difference in BSI between birds that breed at high and low elevations was not significant. According to Bergman's rule, it is expected that at high elevations, individuals will have a larger body size because it allows them to face the low environmental temperatures present at high elevations better due to a higher mass/surface area ratio (Meiri & Dayan, 2003). Moreover, it has also been suggested that larger individuals might cope with altitudinal migration better because they would be more resistant to the harsh weather conditions and lower food abundance present at high elevations (Ketterson & Nolan, 1976). In line with this, our results suggest that the difference in temperature between elevations may influence the body size variation in the center of Chile but not in the south of Chile. Moreover, because in the south the birds that migrated were not larger than those that were at high elevation year‐round, our data cannot support the body size hypothesis.

Our results also showed that ROS and TAC levels were significantly higher in the center than south of Chile. Diverse studies agree that high environmental temperatures increase ROS production (Gonzalez‐Rivas et al., 2020; Mujahid et al., 2007), so it is expected that at lower latitudes ROS and TAC levels will be higher than high latitude. Although, the most birds showed a balance between ROS levels and TAC, the birds that breed at low elevations in the Center of Chile showed significantly higher unbalance ROS/TAC indicating oxidative stress. This unbalance ROS/TAC might be result of the long exposure to extremely high environmental temperatures and the absence of water product of a long drought (>10 years) as a consequence of climatic change (Burger et al., 2018), which may be environmental conditions that overcome the physiological tolerance limits of these birds. In line with this, a recent laboratory study performed in individuals of *Z*. *capensis* from this location, found lower TAC values in birds acclimated to 27°C in comparison to those acclimated to 17°C (Sabat et al., 2019). This suggests that despite the high resistance to oxidative stress observed in birds, it is possible that in this location they have reached a state that is beyond their capacity to counter oxidative damage (Bozinovic & Pörtner, 2015). The climatic variability hypothesis predicts that individuals experiencing a low variation in ambient temperatures should be more affected by both increased and extreme ambient temperatures because their thermal tolerance variation would be smaller than in those individuals living under more variable temperature conditions (Janzen, 1967). This hypothesis may explain why at high elevations, the birds did not show high ROS levels, similar to individuals breeding at low elevations in the center of Chile. However, evidence also shows that high metal levels are directly linked to high ROS levels (Ercal et al., 2001; Koivula & Eeva, 2010). Indirectly, metals may also affect the antioxidant richness present in the food of birds, altering ROS levels (Eeva et al., 2005, 2008). Thus, it is important to assess metal presence and food quality at this study site to clearly understand the cause of the higher oxidative damage in these birds.

Our findings also shown that birds breeding at high elevations exhibited lower exploratory diversity (i.e., slow exploring) compared to those breeding at low elevations (i.e., fast exploring).

The slow exploration behaviors have been linked to higher accuracy in obtaining information (Sih & del Giudice, 2012) and high neophobia toward novel objects (Verbeek et al., 1994). Moreover, slow‐exploring individuals seem to be more flexible in their foraging routine (Marchetti & Drent, 2000), and they might learn better in unpredictable environments than fast‐exploring individuals (Dougherty & Guillette, 2018; Guillette et al., 2010; Kozlovsky et al., 2015). This suggests that slow‐exploring individuals might cope better with the more unpredictable environment present at high elevations because under this condition, reliable information, and greater flexibility would be required to make decisions at low cost. In line with this, a previous study performed in *Poecile gambeli* reporting similar results to those presented here suggested that differences in spatial memory and cognition between birds that breed at high and low elevations may be linked to differences in exploratory behavior (Kozlovsky et al., 2014). However, it is important to note that a previous study of *Z*. *capensis* performed in the center of Chile with data collected during 2013 showed a patter inverse to those reported here, suggesting that exploratory behavior is a context‐dependent trait and may vary from year to year according to the local availability of resources (Dingemanse et al., 2004; Dingemanse & Réale, 2005).

Furthermore, we found that TAC values and exploratory diversity were negatively associated only in birds that breed at high altitudes in southern Chile, suggesting that a decrease in exploratory behavior may favor the ROS/TAC balance, as a result of lower activity levels (Costantini, 2008). However, the fact that this association is lacking in other populations suggests, the links between TAC and exploratory behavior may be context dependent (Costantini & Verhulst, 2009). Interestingly, we also found a direct relationship between oxidative stress and exploratory diversity only in birds breeding at low elevations in the Center of Chile, where the environmental mean and maximum temperatures are higher than those of the other breeding sites. In line with this, a previous study about physiological and behavioral traits in *Z*. *capensis* showed higher exploratory behavior scores and a positive relation between exploratory behavior and metabolic rate, in birds from a similar Mediterranean site (Maldonado, van Dongen, et al., 2012). The ‘Extended’ pace of life hypothesis predicts that individuals that fast‐exploring should show high‐metabolic rate (POLS; Careau et al., 2008; Réale et al., 2010), and hence, metabolic rate might be a possible mechanism behind link between exploratory behavior and oxidative stress in this study population (Arnold et al., 2015). Alternatively, it has been suggested that chronic levels of corticosterone (CORT) increase oxidative damage in birds (Lin et al., 2004). However, a recent study that compared baseline CORT levels of breeding rufous‐collared sparrows present at low elevation (same study site as that of this study) with breeding individuals present at high elevation in the center of Chile showed that both populations have similar basal CORT levels, suggesting that these birds do not show signals of chronic stress (Poblete et al., 2020).

Finally, although there is a considerable debate regarding the effects of environmental temperature on oxidative stress in endothermic animals, recent studies revealed that dehydration and temperature may influence oxidative status, yielding an elevated antioxidant response, and/or oxidative damage (Jacobs et al., 2020; Navarrete et al., 2021; Sabat et al., 2019). Thus, we propose that the links between exploratory behavior and oxidative stress might arise in populations exposed to extreme environmental temperatures and/or dehydration, because under these conditions the capacity to counter the oxidative damage resulting from high‐activity levels might be affected by energetic constraints.

In summary, our results indicate that both altitudinal migration patterns and oxidative status of mountain birds were significantly influenced by the latitude of breeding sites, whereas exploratory behavior exhibited a significant association with elevation. Notably, we observed that fast‐explorer birds inhabiting low elevations in the center of Chile displayed higher levels of oxidative damage in comparison to their slow‐explorer counterparts. These outcomes underscore the possibility of local adaptations in the studied populations, which may have evolved in response to diverse local environmental conditions. Understanding how populations in mountain ecosystems have adapted to their unique environmental conditions, can help us predict how they will respond to challenges stemming from anthropogenic activities, such as climate change, habitat fragmentation, or invasive species. This knowledge is key to designing effective conservation strategies for maintaining the biodiversity and ecological function of mountain ecosystems.

## AUTHOR CONTRIBUTIONS

**Carolina Contreras:** Methodology (equal); writing – review and editing (equal). **Carolina Fernández:** Methodology (equal). **Cristian R. Flores:** Methodology (equal). **Patricia Vega:** Methodology (equal). **Miguel Ávila:** Methodology (equal). **Pablo Sabat:** Formal analysis (supporting); resources (supporting); supervision (lead); writing – review and editing (equal). **Yanina Poblete:** Conceptualization (lead); data curation (lead); formal analysis (lead); funding acquisition (lead); investigation (lead); methodology (equal); project administration (lead); resources (lead); writing – original draft (lead); writing – review and editing (equal).

## FUNDING INFORMATION

Funding was provided by grants from ANID‐FONDECYT 3190111 to YP. We thank funding ANID PIA/BASAL FB0002.

## Supporting information


Appendix S1

## Data Availability

The datasets produced and evaluated during the present study have been archived in the Dryad repository and can be accessed through the following DOI: https://doi.org/10.5061/dryad.0rxwdbs4t.
