# Supplementary material for: Geographic variation in the altitudinal migration patterns, body size, oxidative status and exploratory behavior in a neotropical bird
Source: Ecol Evol. 2023 Mar 26;13(3):e9941. doi: 10.1002/ece3.9941 (PMC10040957; doi:10.1002/ece3.9941)
Supplement: Supplementary file 1 — Appendix S1 [file ECE3-13-e9941-s001.docx]

**Running title: Altitudinal migration, physiology and behavior**

**Supplementary material**

**Geographic variation in the altitudinal migration patterns, body size, oxidative status and exploratory behavior in a neotropical bird.**

**Yanina Poblete^1,2,3*^, Carolina Contreras^1,2,3^, Carolina Fernández^4^, Cristian R. Flores^5^, Patricia Vega^6^, Miguel Ávila^1^ and Pablo Sabat^2,3^**

^1^ NIAVA: Núcleo de Investigaciones Aplicadas en Ciencias Veterinarias y Agronómicas, Instituto de Ciencias Naturales, Facultad de Medicina Veterinaria y Agronomía, Universidad de Las Américas, Campus Providencia, Manuel Montt 948, Santiago, Chile.

^2^ Departamento de Ciencias Ecológicas, Facultad de Ciencias, Universidad de Chile, Santiago, Chile.

^3^ Center of Applied Ecology and Sustainability (CAPES), Santiago, Chile.

^4^ Facultad de Ciencias Forestales y Conservación de la Naturaleza, Universidad de Chile, Santiago, Chile.

^5^ Liceo Armando Robles Rivera, Arauco 474, Valdivia, Chile.

^6^ Master of Conservation Science, University of Queensland, Australia.

**Corresponding author**: [yanina.poblete@udla.cl](mailto:yanina.poblete@udla.cl); ORCID: 0002-1378-9341

**Methods of molecular sexing**

**DNA extraction**

DNA was extracted from dried blood spots using Dried Blood Spot DNA Isolation Kit (Norgen Biotek Corp) following manufacturer's protocol. DNA yield was determined by measuring the concentration of DNA in the eluate by its absorbance at 260 nm using Picodrop spectrophotometer (Pico200, Picodrop Limited).

**Polymerase Chain Reaction for sexing birds**

All PCR reactions were performed in 10 ul volumes using 15 ng of DNA, 1.25 U Platinum Taq DNA Polymerase with 1x of PCR buffer (Invitrogen), 0.25 mM dNTPs, 1.75 mM MgCl2, and 1 uM of primers 2550F (5'-GTTACTGATTCGTCTACGAGA-3') and 2718R (5'-ATTGAAATGATCCAGTGCTTG-3'). Finally, the PCR reaction with DNA as template was carried out with the following conditions, 94°C for 2 min, 45 cycles of denaturation at 94°C for 30 sec, annealing for 45 sec at 48°C and extension at 72°C for 45 sec, followed by final extension at 72°C for 10 min. PCR products were separated in 1% agarose gels (FMC Bioproducts, Seakem), run in standard TAE buffer, and visualized by Gel Red (Biotium) staining.

**Table S1.** Results from Principal Component Analyses (PCA) on morphological measures recorded in Rufous-collared sparrow (n=72). PC loadings, eigenvalues, and estimations of explained variance are presented.

| **Morphological**  **measures** | **Component 1**  **Body Condition Index** |
| --- | --- |
| Bill length (mm) | -0.257 |
| Tarsus length (mm) | -0.362 |
| Wing length (mm) | -0.565 |
| Tail length (mm) | -0.495 |
| Body mass (grs) | -0.487 |
| **Eigenvalue** | **1.444** |
| **Variance explained** | **0.416** |

**Table S2.** Results from general lineal models for testing relationship between individual variation in TAC, TBARS, oxidative status and exploratory behavior with body size (PCA-1 BCI) and sex in Rufous-collared sparrow (n=72). SE: standard error; L/U 95% CI = lower/upper bound.

| **TAC** | **Estimate** | **SE** | **L 95% CI** | **U 95% CI** |
| --- | --- | --- | --- | --- |
| Intercept | 1.16 ^e+01^ | 7.32 ^e+01^ | **0.010** | **0.013** |
| Body size | -0.045 | 0.357 | -0.001 | 0.001 |
| Sex^a^ | 1.349 | 1.170 | -0.001 | 0.001 |
| **TBARS**  (*F* =0.61; *df.* = 41; *p* = 0.61) | **Estimate** | **SE** | **L 95% CI** | **U 95% CI** |
| Intercept | -412717 | 27505 | **-4676680.44** | **-357752.92** |
| Body size | -10350 | 14111 | -38548.63 | 17848.74 |
| Sex^a^ | 115149 | 44453 | **26317.07** | **203981.73** |
| **Oxidative status**  (*F* =1.36; *df.* =77; *p* =0.26) | **Estimate** | **SE** | **L 95% CI** | **U 95% CI** |
| Intercept | -397.447 | 30.529 | **-458.455** | **-336.439** |
| Body size | -7.478 | 15.074 | -37.601 | 22.644 |
| Sex^a^ | 8.783 | 47.984 | -87.104 | 104.67 |
| **Exploratory behavior**  (*F* =1.36; *df.* =77; *p* =0.26) | **Estimate** | **SE** | **L 95% CI** | **U 95% CI** |
| Intercept | 1.196 | 0.047 | 1.103 | 1.290 |
| Body size | -0.069 | 0.024 | **-0.116** | **-0.022** |
| Sex^a^ | -0.113 | 0,077 | -0.267 | 0.041 |

^a^Parameter estimates and SE were estimated relative to ‘Male’ level in variable ‘Sex’.

**Table S3.** Pairwise comparisons of means populations for Body size, TAC, TBARS, oxidative status and exploratory behavior among study sites, altitude and latitude. P values <0.05 in bold.

| Effect on Body Size  Study sites | Study sites | | |
| --- | --- | --- | --- |
|  | Lagunillas | Rinconada | Conguillío |
| Rinconada | **0.002** | - | - |
| Conguillío | 0.344 | **0.03** | - |
| Rucamanque | **0.012** | 0.59 | 0.14 |
| Altitudes | Low | Latitudes | South |
| High | **0.001** | Center | 0.76 |
| Effect on TAC | Study sites | | |
| Study sites | Lagunillas | Rinconada | Conguillío |
| Rinconada | 0.42 | - | - |
| Conguillío | **4.6 e-06** | **8.9e-05** | - |
| Rucamanque | **8.3e-07** | **1.9e-05** | 0.73 |
| Altitudes | Low | Latitudes | South |
| High | 0.43 | Center | **2.5e-09** |
| Effect on TBARS | Study sites | | |
| Study sites | Lagunillas | Rinconada | Conguillío |
| Rinconada | **0.01** | - | - |
| Conguillío | **5.5e-06** | **3.5e-10** | - |
| Rucamanque | **0.001** | **1.1e-07** | 0.11 |
| Altitudes | Low | Latitudes | South |
| High | **0.04** | Center | **5.8e-10** |
| Effect on TBARS/TAC | Study sites | | |
| Study sites | Lagunillas | Rinconada | Conguillío |
| Rinconada | **0.0003** | - | - |
| Conguillío | 0.21 | **0.02** | - |
| Rucamanque | **0.01** | 0.35 | 0.16 |
| Altitudes | Low | Latitudes | South |
| High | **0.0004** | Center | 0.7 |

| Effect on Body size  Effect on  Exploratory diversity | Study sites  Study sites | | |
| --- | --- | --- | --- |
| Study sites | Lagunillas | Rinconada | Conguillío |
| Rinconada | **0.004** | - | - |
| Conguillío | 0.95 | **0.008** | - |
| Rucamanque | **0.005** | 0.99 | **0.009** |
| Altitudes | Low | Latitudes | South |
| High | **0.0001** | Center | 0.88 |


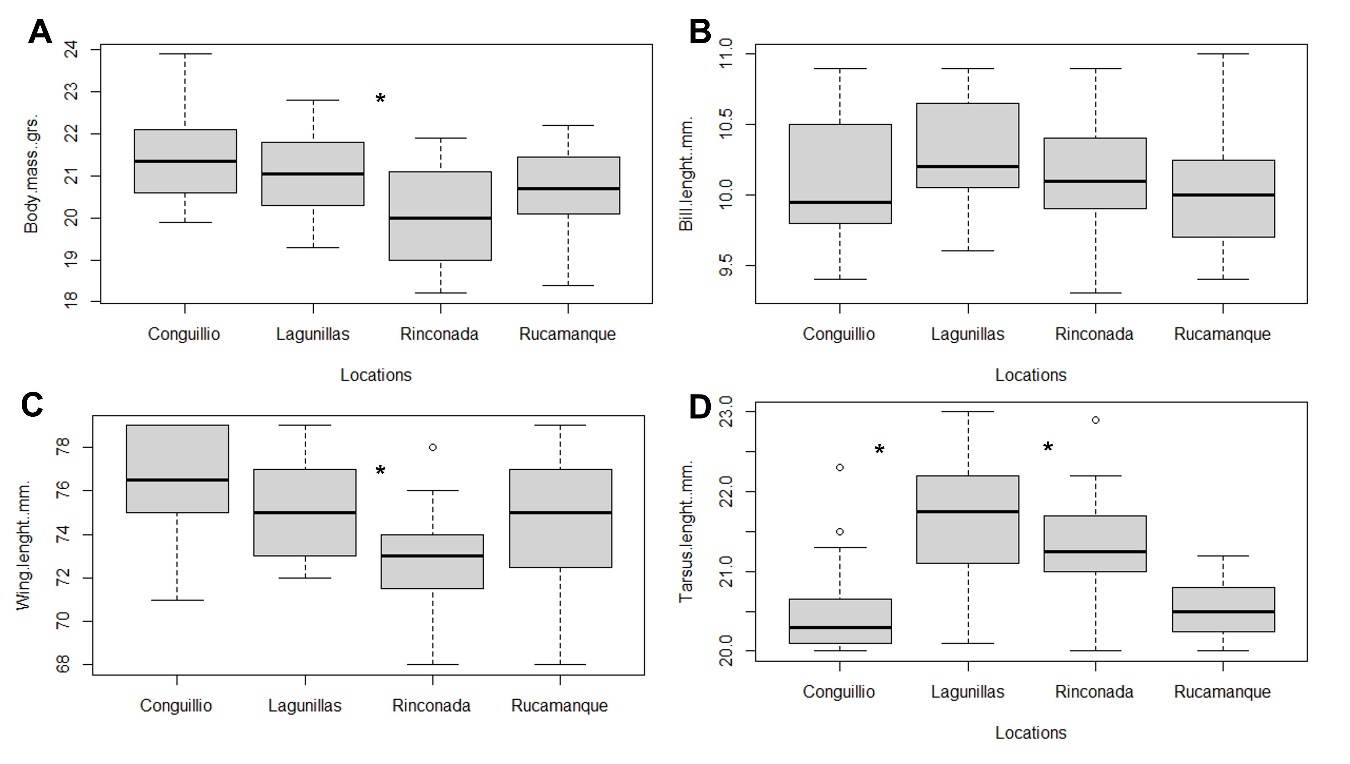


Figure S1. Morphometric measures in rufous collared sparrows (n=72). The bold horizontal line inside the box represents the median score for body mass (A) bill (B), wing (C) and tarsus length (D), respectively. Vertical lines indicate standard error and asterisks indicate significant differences among groups.
